# Correction to T‐cell derived extracellular vesicles prime macrophages for improved STING based cancer immunotherapy

**DOI:** 10.1002/jev2.12372

**Published:** 2023-10-19

1

Aida S. Hansen, Lea S. Jensen, Kristine R. Gammelgaard, Kristoffer G. Ryttersgaard, Christian Krapp, Jesper Just, Kasper L. Jønsson, Pia B. Jensen, Thomas Boesen, Mogens Johannsen, Anders Etzerodt, Bent W. Deleuran, Martin R. Jakobse

In the original article, author Mogens Johannsen's name was misspelled as Mogens Johansen. This has been corrected in the online version of the article.

We apologize for this error.

